# Announcement

**Published:** 1893-05

**Authors:** 


### ANNOUNCEMENT.

It affords us much pleasure to announce that we have added to our editorial staff, Dr. Louis H. Jones, whose well known ability as a writer and teacher is such as to require no introduction at our hands. Dr. Jones has, for the past three years, ably filled the chair of Chemistry in the Atlanta Medical College.

---

We have just been advised of the death of Dr. J. E. Roach, of Sipe Springs, Texas, on April 10th.

Dr. Roach was a Georgian, and practiced in this city two years after graduation. He was engaged in active practice for thirteen years in the above named place and was held in high esteem by the profession and laity.

---

We have been coming out about the middle of the month. Commencing with the June issue we will be out on the first of each month.

---

A bill now before the Pennsylvania Legislature forbids the exhibition of monstrosities at public places like the Museum.

---

SANITATION AND THE COMMUNION TABLE.—The agitation concerning the advisability of doing away with the communion cup in church services, and substituting therefor individual cups for each member, is bearing fruit. Our friend, Dr. A. J. Longfellow, of Fostoria, O., at the last quarterly conference of the M. E. Church, introduced the following resolution : "Resolved, That the church purchase four hundred little wine-glasses and each communicant receive the wine out of a glass that no other person has used, and the bread be passed on baskets or plates, and that it be not handled or broken by the preacher."—*Cincinnati Lancet Clinic.*

